# Influence of Acute Exposure to High Altitude on Basal and Postprandial Plasma Levels of Gastroenteropancreatic Peptides

**DOI:** 10.1371/journal.pone.0044445

**Published:** 2012-09-06

**Authors:** Rudolf L. Riepl, Rainald Fischer, Hubert Hautmann, Gunther Hartmann, Timo D. Müller, Matthias Tschöp, Marcell Toepfer, Bärbel Otto

**Affiliations:** 1 Department of Medicine, Gastroenterology, Kreiskrankenhaus Erding, Erding, Germany; 2 Medizinische Klinik und Poliklinik IV, Klinikum der Universität München – Campus Innenstadt, Munich, Germany; 3 Department of Medicine, Technische Universität München, Munich, Germany; 4 Clinical Pharmacology, University Hospital Bonn, Bonn, Germany; 5 Institute for Diabetes and Obesity, Helmholtz Centre Munich, Department of Medicine, Technische Universität München, Munich, Germany; 6 General Practice, Murnau, Germany; University of Cordoba, Spain

## Abstract

Acute mountain sickness (AMS) is characterized by headache often accompanied by gastrointestinal complaints that vary from anorexia through nausea to vomiting. The aim of this study was to investigate the influence of high altitude on plasma levels of gastroenteropancreatic (GEP) peptides and their association to AMS symptoms. Plasma levels of 6 GEP peptides were measured by radioimmunoassay in 11 subjects at 490 m (Munich, Germany) and, after rapid passive ascent to 3454 m (Jungfraujoch, Switzerland), over the course of three days. In a second study (n = 5), the same peptides and ghrelin were measured in subjects who consumed standardized liquid meals at these two elevations. AMS symptoms and oxygen saturation were monitored. In the first study, both fasting (morning 8 a.m.) and stimulated (evening 8 p.m.) plasma levels of pancreatic polypeptide (PP) and cholecystokinin (CCK) were significantly lower at high altitude as compared to baseline, whereas gastrin and motilin concentrations were significantly increased. Fasting plasma neurotensin was significantly enhanced whereas stimulated levels were reduced. Both fasting and stimulated plasma motilin levels correlated with gastrointestinal symptom severity (r = 0.294, p = 0.05, and r = 0.41, p = 0.006, respectively). Mean O_2_-saturation dropped from 96% to 88% at high altitude. In the second study, meal-stimulated integrated ( = area under curve) plasma CCK, PP, and neurotensin values were significantly suppressed at high altitude, whereas integrated levels of gastrin were increased and integrated VIP and ghrelin levels were unchanged. In summary, our data show that acute exposure to a hypobaric hypoxic environment causes significant changes in fasting and stimulated plasma levels of GEP peptides over consecutive days and after a standardized meal. The changes of peptide levels were not uniform. Based on the inhibition of PP and neurotensin release a reduction of the cholinergic tone can be postulated.

## Introduction

Acute mountain sickness (AMS), a syndrome often observed in newcomers at high altitude, is characterized by headache often accompanied by gastrointestinal symptoms like anorexia, nausea, and even vomiting [1], [2]. The molecular underpinnings regulating these complex symptoms are not well understood, but the autonomic nervous system and endocrine mechanisms are likely being involved. Gastroenteropancreatic (GEP) peptides regulate gastrointestinal functions by acting as neurotransmitters of the autonomic and enteric nervous system as well as hormones via the circulation [3], [4]. Cholecystokinin, for example, plays a role as a satiety signal whereas ghrelin is stimulating food intake while decreasing energy expenditure. Several lines of evidence indicate that energy deficit at high altitude results in a loss of body mass in both healthy [5] and obese [6] subjects. Accordingly, based on the key role of GEP peptides in systemic energy metabolism control we hypothesized that the release of such peptides may be affected in AMS, e.g. by causing a decrease of appetite and food intake. [7].

So far only few and partly conflicting data are available concerning the influence of high altitude or experimental hypoxemia on the release of GEP peptides in humans [8]–[16] and animals [17]–[22]. In man, no more than two peptides were measured simultaneously and data on the response to a standardized physiological stimulus are not available.

Accordingly, the aim of this study was to assess pre- and postprandial levels of up to seven GEP peptides at high altitude compared to sea levels and to assess the potential association of these peptides with AMS symptoms.

## Materials and Methods

In the first study, blood was drawn from an antecubal vein from eleven young healthy subjects (1 female, 10 male) at baseline (490 m, Munich, Germany) and after rapid passive ascent by train to 3454 m (research laboratory at Jungfraujoch, Switzerland). The subjects reached this altitude at 6 p.m. and stayed there for 3 nights. The study subjects had no or very little experience at high altitude and some individuals have previously been described elsewhere [23].

**Table 1:** Influence of rapid exposure of 11 subjects to high altitude on fasting plasma concentrations of gastroentero-pancreatic peptides and on acute mountain sickness (AMS) score at 8 a.m.

FASTING	Munich	Jungfrau- Joch (3454 m )			Munich
Morning 8 AM	(490 m)	14 hours	38 hours	62 hours	(490 m)
AMS score	0	8.5±2.3**	9.0±2.6**	6.4±1.6**	0
CCK (pmol/L)	2.4±1.6	2.4±1.6	1.2±1.3*	1.8±1.6	2.0±1.0
Gastrin (pg/mL)	64.9±30	93.6±70*	73.9±28	73.3±32	61.6±27
Motilin (pmol/L)	70.5±10	70.8±10	75.5±9	78.0±12*	74.3±14
PP (pmol/L)	16.7±13	14.5±11	9.0±5*	9.9±7**	11.4±7*
Neurotensin (pmol/L)	8.4±4.6	11.8±4.6*	9.3±3.9	8.6±3.0	9.1±3.9
VIP (pmol/L)	5.9±2.6	5.6±2.3	4.7±3.0	5.0±1.0	5.5±1.6

Asterisks indicate significant changes as compared to baseline levels in Munich before the ascent (* = p<0.05; ** = p<0.01).

**Table 2:** Influence of rapid exposure of 11 subjects to high altitude on stimulated plasma concentrations of gastroentero-pancreatic peptides and on acute mountain sickness (AMS) score at 8 p.m.

STIMULATED	Munich	Jungfrau- Joch (3454 m )			Munich
Evening 8 PM	(490 m)	2 hours	26 hours	50 hours	(490 m)
AMS score	0	6.0±1.6**	5.7±1.6**	5.1±1.0**	0
CCK (pmol/L)	4.7±2.6	5.2±2.3	2.6±2.0*	2.4±1.3*	3.2±2.6
Gastrin (pg/mL)	87.4±31	95.0±43	114.4±65*	92.5±49	80.7±27
Motilin (pmol/L)	70.3±10	69.7±11	74.5±12*	75±11*	70.5±10
PP (pmol/L)	87.5±75	67.0±47	60.9±38*	42.0±36*	65.7±50
Neurotensin (pmol/L)	24.9±9.2	25.1±11.5	14.8±3.3*	16.2±3.3*	18.7±9.2
VIP (pmol/L)	4.4±1.3	5.0±2.0*	4.5±1.3	4.1±2.0	4.7±1.6

Asterisks indicate significant changes as compared to baseline levels in Munich before the ascent (* = p<0.05; ** = p<0.01).

**Figure 1:**
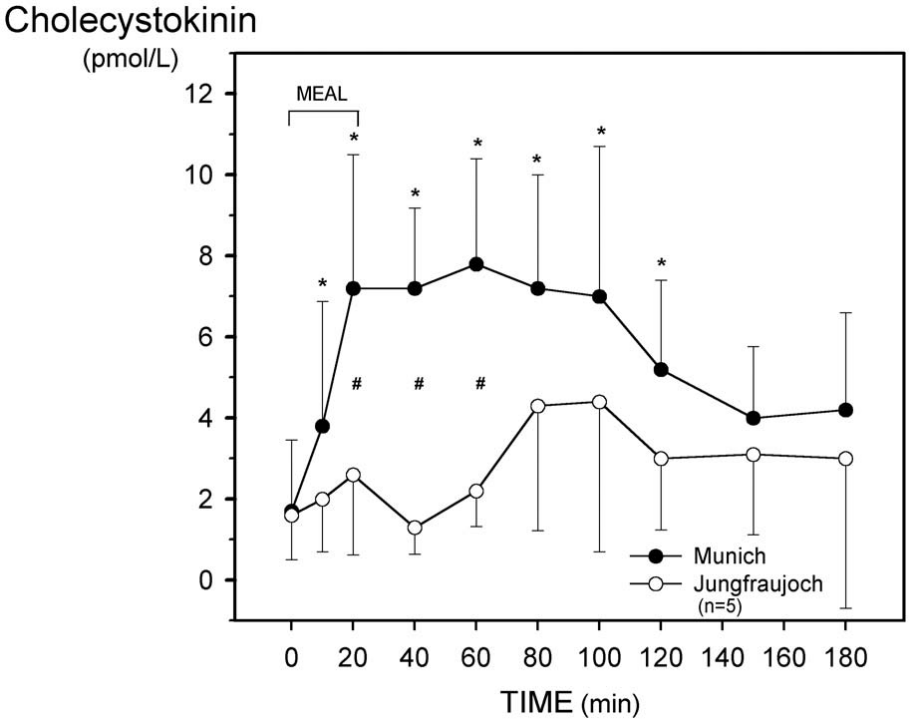
Effect of acute exposure to high altitude (Munich, 490 m, to Jungfraujoch, 3454 m) on postprandial plasma levels of cholecystokinin (CCK). Asterisks (*) indicate a significant increase (p<0.03) as compared with the preprandial values at 0 min and crosses (#) mean a significant (p<0.03) difference between the respective values at the two sea levels.

AMS symptoms were monitored twice daily at 8 a.m. and 8 p.m. using the Lake Louise self report questionnaire recording headache, gastrointestinal symptoms (nausea, vomiting), fatigue, dizziness, and sleeplessness [24]. Each of the five symptoms was rated on a scale of 0 (none) to 3 (severe). A total score of 3–5 describes mild and 6 and more moderate to severe AMS with a maximal score of 15. The AMS sum score and each symptom score was correlated to GEP peptide levels.

Capillary blood gas analysis was performed in the resting state in Munich, within 2 hours after reaching Jungfraujoch in the evening, and 14 hours thereafter in the morning.

Venous blood samples of 10 ml were drawn into ice-chilled EDTA tubes at 8 a.m. (fasting) and 8 p.m. (stimulated, i.e. 2 hours after dinner; food was given at libitum and not standardized) at Munich, every day at Jungfraujoch (fasting time points 14, 38, 62 hours and postprandial time points 2, 26, and 50 hours after arrival at high altitude, respectively), and again at Munich two weeks after return. Plasma was obtained immediately by centrifugation and stored at −20°C until radioimmunoassays were performed to determine the concentrations of cholecystokinin (CCK), gastrin, motilin, pancreatic polypeptide (PP), neurotensin, and vasoactive intestinal peptide (VIP).

In the second study, which was performed 15 months later, participated five young healthy male subjects, including three individuals of the first study. They had more experience at high altitude, but none had an overnight stay over 2000 m above sea level for the last six months to exclude adaptation processes. Rapid passive ascent by train was performed comparable to study 1. Capillary oxygen saturation was monitored at Munich and at Jungfraujoch. The subjects fasted overnight and drank a standardized liquid meal (500 ml Fresubin®, Fresenius, Bad Homburg, Germany) supplemented with 15 ml corn oil (12.6% protein, 45.6% carbohydrate, 41.7% fat = 586 kcal) in the morning at baseline (490 m, Munich) and in the first morning 18 to 20 hours after arrival by train at Jungfraujoch. Based on weight the liquid meal comprised of 18 g protein, 65 g carbohydrates and 26.9 g fat. A meal with a relatively high fat content was chosen to induce a robust stimulus of GEP peptide release. Venous blood samples of 10 ml were drawn into ice-chilled EDTA tubes before and at several time points (10, 20, 40, 60, 80, 100, 120, 150, and 180 min) after ingestion of the meal. The tubes were centrifuged immediately and the plasma was stored at −20°C until measurement of GEP peptides and ghrelin using radioimmunoassays.

The study was approved by the Ethics Committee of the Medical Faculty of the University of Munich. Each subject gave written informed consent and the study was carried out according to the declaration of Helsinki.

**Table 3:** Influence of high altitude (Munich, 490 m, to Jungfraujoch, 3454 m) on postprandial release of gastroenteropancreatic (GEP) peptides after a standardized liquid meal.

	MUNICH (490 m)			JUNGFRAUJOCH (3454 m)			
GEP PEPTIDES	basal	postprandial (0–180 min)	incremental response	basal	postprandial (0–180 min)	incrementalresponse	difference of incremental response
**CCK** (pmol⋅180 min/L)	302±297	1066±354	764±323*	302±178	523±275	221±345	p = 0.025
**Gastrin** (pg⋅180 min/mL)	21960±7535	24444±7515	2484±2065*	23652±8529	27625±8459	3973±2048*	p = 0.025
**Motilin** (pmol⋅180 min/L)	12600±2356	10931±1790	−1669±1036*	12564±4236	10491±3187	−2073±1372*	n.s.
**PP** (pmol⋅180 min/L)	338±169	5224±2171	4886±2050*	212±46	2055±1320	1843±1322*	p = 0.025
**Neurotensin** (pmol⋅180 min/L)	1692±965	3911±974	2219±1097*	5760±1097	6167±1493	407±1150	p = 0.025
**VIP** (pmol⋅180 min/L)	1206±327	1185±266	−21±347	1263±264	1595±486	332±512	n.s.
**Ghrelin** (pmol⋅180 min/L)	5731±1971	4566±1177	−1165±805*	4852±1502	3201±926	−1651±611*	n.s.

Asterisks (*) indicate a significant (p<0.03) change of integrated peptide values ( = area under curve) as compared to the preprandial values. In the last column p - values mean a significant difference of incremental integrated peptide responses between Munich and Jungfraujoch. Incremental response represents the total postprandial peptide release.

### Radioimmunoassays

Plasma concentrations of GEP peptides were determined by radioimmunoassays (RIA). The assays for CCK [25], neurotensin [26], PP [27], and VIP [28] were established and evaluated in our laboratory of gastroenterology. The respective coefficients of intra-assay variation were 5.6, 9.8, 11.1, and 13.3%, and those of inter-assay variation were12.3, 14.0, 13.1, and 15.3%. The CCK-RIA detects all biologically active, sulphated molecular forms of CCK with <1% cross-reactivity to sulphated gastrins. The neurotensin RIA recognizes the intact molecule (neurotensin_1–13_) to 100% and neurotensin_1–11_ to 70%. The other RIAs were commercially available kits (Motilin: Euro-Diagnostica, Malmö, Schweden; Gastrin: GASK-PR, Isotopen Diagnostik CIS, Dreieich, Germany; Ghrelin: Phoenix Pharmaceuticals, Belmont, CA, USA). Plasma concentrations of peptides were not corrected for possible shifts of plasma volume at high altitude since the observed changes of hemoglobin and hematocrit were only minimal in this short time period (personal observation).

**Figure 2:**
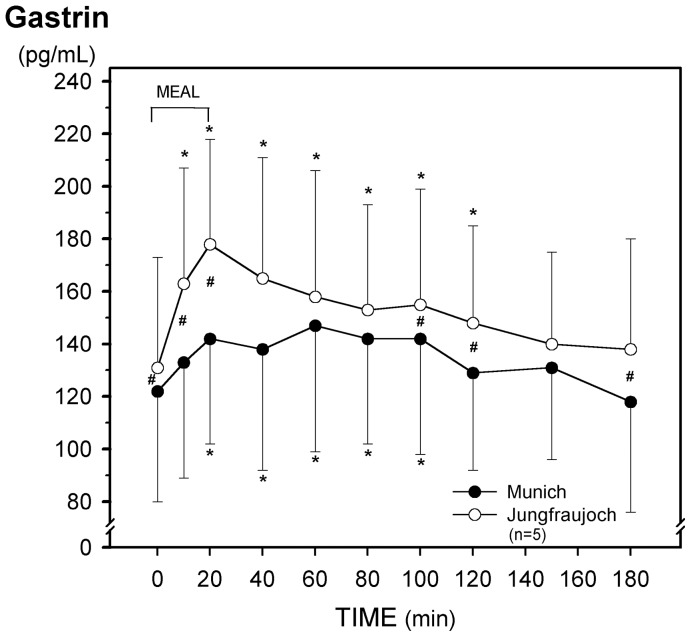
Effect of acute exposure to high altitude (Munich, 490 m, to Jungfraujoch, 3454 m) on postprandial plasma levels of gastrin. Asterisks (*) indicate a significant increase (p<0.03) as compared with the preprandial values at 0 min and crosses (#) mean a significant (p<0.03) difference between the respective values at the two sea levels.

### Calculations and Statistics

Non parametric tests were used as our data do not follow normal distribution due to the low number of subjects. In the first study, changes of peptide levels were analyzed by comparing the values at baseline (Munich) with those after ascent to Jungfraujoch by using the Friedman two-way analysis of variance. Correlations between AMS symptoms and plasma concentrations of GEP peptides were determined using linear regression analysis.

In the second study, meal-stimulated peptide values were compared with preprandial values using the Friedman two-way analysis of variance. Integrated peptide values were calculated as the areas under the concentration curves in order to compare the “total” peptide release. The integrated incremental responses (Δ) were obtained by subtraction of the preprandial levels. Integrated incremental responses at Jungfraujoch and at Munich were compared and analyzed with the Wilcoxon test for paired data. P < 0.05 was considered statistically significant. All values are given in mean ± S.D.

**Figure 3:**
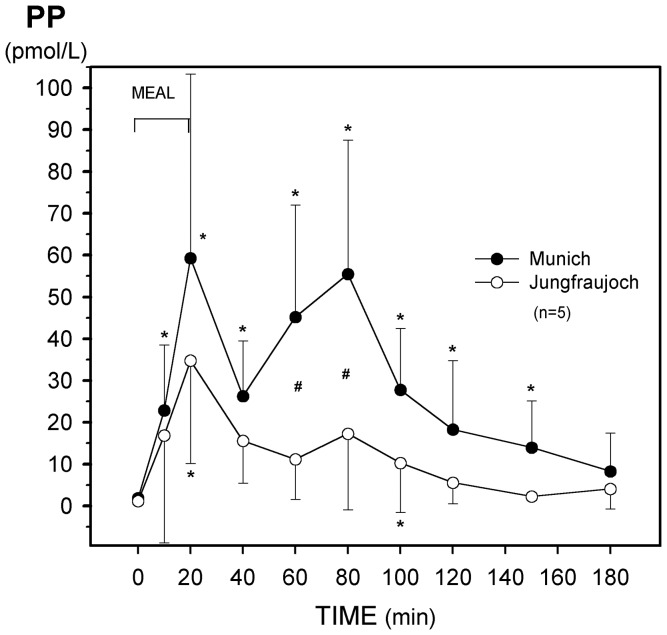
Effect of acute exposure to high altitude (Munich, 490 m, to Jungfraujoch, 3454 m) on postprandial plasma levels of pancreatic polypeptide (PP). Asterisks (*) indicate a significant increase (p<0.03) as compared with the preprandial values at 0 min and crosses (#) mean a significant (p<0.03) difference between the respective values at the two sea levels.

**Figure 4:**
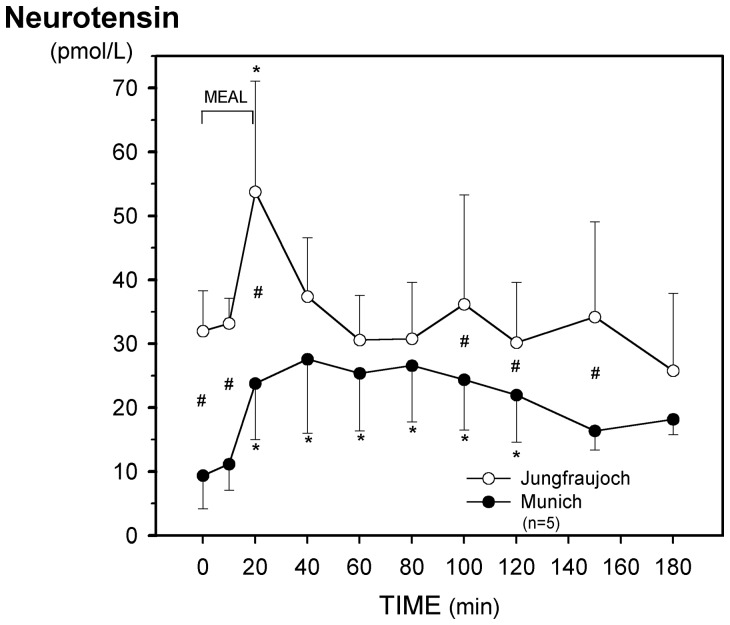
Effect of acute exposure to high altitude (Munich, 490 m, to Jungfraujoch, 3454 m) on postprandial plasma levels of neurotensin. Asterisks (*) indicate a significant increase (p<0.03) as compared with the preprandial values at 0 min and crosses (#) mean a significant (p<0.03) difference between the respective values at the two sea levels.

## Results

Capillary oxygen pressure pO_2_ and oxygen saturation (SaO_2_) significantly decreased from 91.9±2 mmHg and 96.0±2% in Munich to 48.5±8.2 mmHg and 87.4±4.3% during the first evening after ascent to Jungfraujoch (3454 m), and 55.6±3.3 mmHg and 91.9±2.6% the next morning. Hyperventilation caused a significant reduction of capillary pCO_2_ from 39.4±2 mmHg to 34.4±4.2 and 33.0±2.3 mmHg, respectively, and an augmentation of pH from 7.40±0.03 to 7.45±0.03 and 7.44±0.03, respectively.

**Figure 5:**
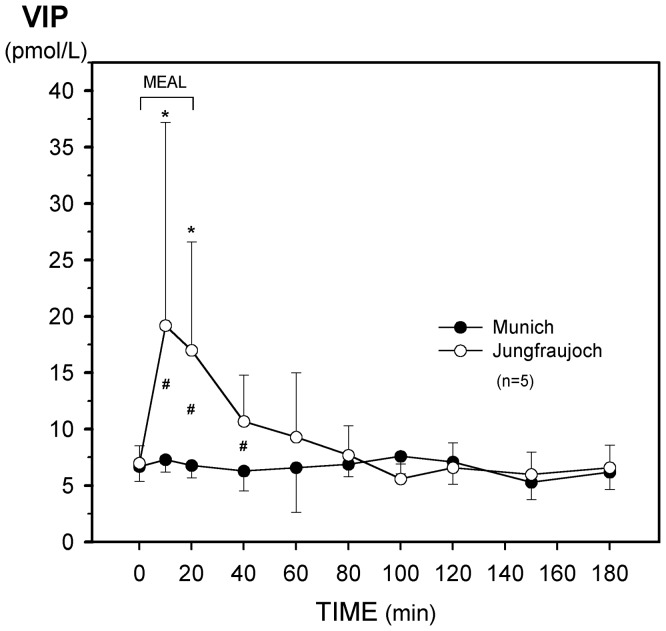
Effect of acute exposure to high altitude (Munich, 490 m, to Jungfraujoch, 3454 m) on postprandial plasma levels of vasoactive intestinal peptide (VIP). Asterisks (*) indicate a significant increase (p<0.03) as compared with the preprandial values at 0 min and crosses (#) mean a significant (p<0.03) difference between the respective values at the two sea levels.

**Figure 6:**
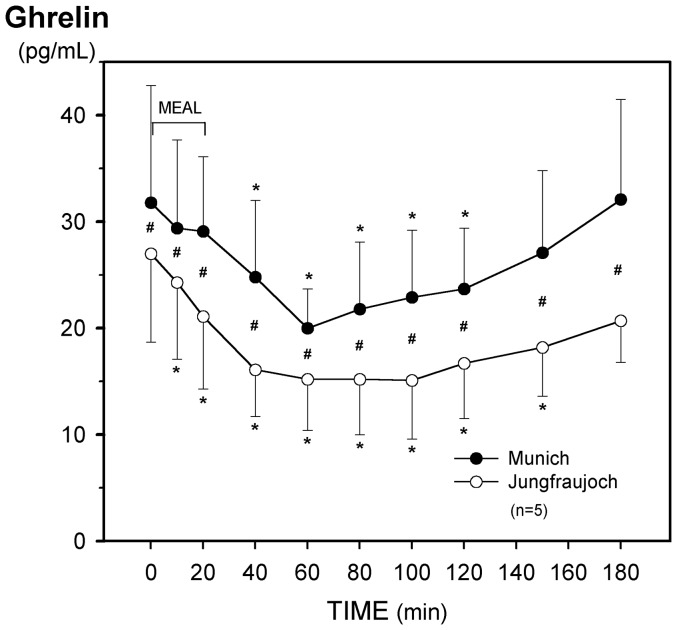
Effect of acute exposure to high altitude (Munich, 490 m, to Jungfraujoch, 3454 m) on postprandial plasma levels of ghrelin. Asterisks (*) indicate a significant decrease (p<0.03) as compared with the preprandial values at 0 min and crosses (#) mean a significant (p<0.03) difference between the respective values at the two sea levels.

In the morning after 38 hours at high altitude the AMS sum score reached its maximum of 9±2.6 points (p<0.01; Table 1). Compared to baseline measures in Munich, fasting plasma concentrations of gastrin and neurotensin were significantly increased after 14 hours, and those of motilin after 62 hours, at Jungfraujoch. In contrast, plasma levels of both CCK and PP were decreased after 38 hours at Jungfraujoch (p<0.05) and those of PP even remained lower after 62 hours (p<0.01). Fasting VIP levels showed a falling tendency without reaching statistical significance (Table 1).

In the evening, the AMS sum score was always lower than in the morning and reached its maximum of 6.0±1.6 points immediately after 2 hours at high altitude followed by a slow decline (p<0.01; Table 2). Stimulated plasma levels of VIP were significantly increased after 2 hours, gastrin and motilin after 26 hours, and motilin also after 50 hours at Jungfraujoch as compared with Munich (p<0.05). In contrast, plasma CCK, PP, and neurotensin were significantly decreased after 26 hours and 50 hours (p<0.05).

A significant correlation was found between plasma motilin and gastrointestinal symptom severity in the morning (r = 0.3, p = 0.05) as well as in the evening (r = 0.44, p = 0.0026). In the evening, plasma motilin correlated also with headaches (r = 0.41, p = 0.006), but not with the AMS sum score. The other peptides measured showed no correlations between plasma levels and the AMS sum score or the score of any of the five symptoms, respectively. Significant correlations were detected in the evening between neurotensin and PP (r = 0.52, p = 0.0003), CCK and PP (r = 0.496, p = 0.0006), and CCK and neurotensin (r = 0.51, p = 0.0004).

In the second study, SaO_2_ dropped from 96.2±1.5% at Munich to 91.2±0.7% the next morning after arrival at Jungfraujoch. The mean AMS sum score of the five subjects reached 2.4±2 points in the morning before intake of the liquid meal.

Plasma levels of CCK showed a significant increase within the first two hours after the standardized liquid meal at Munich, this increase was however almost blunted at Jungfraujoch (Fig. 1). Accordingly, incremental integrated plasma CCK was significantly lower at high altitude (Table 3).

In contrast, for fasting plasma gastrin concentrations, the postprandial increases at several time points and incremental integrated plasma gastrin were significantly higher at Jungfraujoch (Fig. 2, Table 3).

In both studies, plasma levels of motilin were significantly reduced after 40 min to 120 min after intake of the meal, notably, however, without changes in the total area under the curve (Table 3).

Ingestion of the meal caused a biphasic increase of plasma PP levels which was attenuated at Jungfraujoch (Fig. 3). Incremental integrated plasma PP values over 180 min were also suppressed at high altitude (Table 3).

Fasting plasma concentrations of neurotensin were more than three times higher at Jungfraujoch than in Munich. In contrast to the test in Munich, the meal caused only a transient release of neurotensin at high altitude (Fig. 4, Table 3).

Plasma levels of VIP showed a steep postprandial increase at Jungfraujoch whereas no changes were seen in Munich (Fig. 5). Incremental integrated VIP did not differ (Table 3).

Ingestion of the meal reduced plasma ghrelin levels to a nadir after 60 min at both altitudes. Fasting and postprandial ghrelin levels, however, were significantly lower at Jungfraujoch, but incremental integrated ghrelin release did not differ (Fig. 6, Table 3).

## Discussion

In the first study we assessed, over the course of three days, a time profile of plasma GEP peptides in the morning (fasting) and in the evening (stimulated) after acute exposure to a hypobaric hypoxic environment (increase in altitude of almost 3000 m).

Based on the observation that the predominant changes of peptide levels occurred between 14 and 50 hours at high altitude, we applied a standardized test meal (18 to 20 hours after ascent) in the second study. Noteworthy, peptide response and symptom score of the only female participant in study 1 was similar to those observed in the male participants. Accordingly, data obtained from the female participant were not excluded from the analysis.

In the first study, in which the participants had no or very little experience at high altitude, moderate symptoms of AMS were reported in the morning hours after rapid exposure to hypobaric hypoxic conditions. In the second study, the participants hardly developed mild AMS symptoms, thus indicating that the difference in the AMS score might primarily be explained by the lower susceptibility to hypoxic conditions. However, the mean oxygen saturation dropped comparably in both study groups (from approximately 96% to 91%) 14 hours and 18 hours after arrival at high altitude. Several lines of evidence indicate that hypoxemia considerably affects physiological body regulation at high altitude [2]. However, even though gastrointestinal symptoms occur frequently, so far only few studies assessed the influence of hypoxia on gastrointestinal functions and the release of regulatory peptides at high altitudes.

In study 1, plasma levels of CCK were decreased under both fasted and fed conditions after exposure to high altitudes for 38 hours and 26 hours. In line with this observation, in the second study (18 hours at Jungfraujoch) CCK release was almost blunted within the first 60 min after the standardized meal and integrated peptide values remained significantly suppressed up to 180 min. Noteworthy, the decrease in CCK release might be secondary due to an alteration of gastric emptying. Both acceleration (rapid passage of the liquid meal through the upper intestine) and inhibition (delayed appearance of the stimulus in the intestine) of gastric emptying could reduce the contact of chyme with the CCK releasing I-cells in the mucosa of the duodenum and small intestine [29]. Notably, Bailey et al. [8] reported increased level of resting plasma CCK at 5100 m after a several-day trek to Kanchenjunga basecamp. The increase was even more pronounced in subjects with AMS score >3. This controversy to our study might be explained by the more acute exposure of the subjects to hypoxic hypobaric conditions as acute and chronic exposure to hypoxia may provoke different CCK release [9].

CCK is well known for its effect to suppress food intake [29], [30]. Accordingly, in the chronic setting [8] increased CCK release may contribute to the observed anorexia and loss of body weight at high altitude but not in the acute setting where CCK release is reduced.

Interestingly, both fasting and stimulated plasma levels of gastrin increased within 14 to 26 hours after arrival at 3454 m and fell to the normal range over the following days. Noteworthy, this might explain why Gritti et al. [11] observed no change of serum gastrin concentrations after 6 days at 4300 m as compared to sea level. In contrast, dwellers of the Peruvian mountains (residence at 3730 m) are reported to have higher basal gastrin levels compared to those living in Lima (150 m) [12]. One explanation for this might be that basal gastric acid secretion is diminished in subjects permanently living at high altitude, and that the release of gastrin is augmented via a positive feedback mechanism [12], [13]. In line with this assumption, Jó et al. [31] found a lower sensitivity of the gastric parietal cell to intravenous pentagastrin in a group of 17 Andean subjects as compared with a matched group at sea level.

Up to two hours after intake of a mixed meal Jó et al. [12] further observed an exaggerated release of serum gastrin in dwellers of the Peruvian mountains as compared to those living in Lima. Similarily, after drinking a liquid meal, our subjects, who were exposed acutely to a hypobaric hypoxic environment, displayed higher plasma gastrin concentrations and integrated peptide release up to 180 min after the meal. The most likely reason for this finding is that stimulated acid release is attenuated soon after arrival at high altitude as has been shown in dwellers living in the Peruvian mountains [12].

As compared to baseline levels at Munich, fasting plasma levels of motilin were significantly higher after 62 hours at Jungfraujoch whereas stimulated values were significantly higher after 26 hours. Physiologically, motilin is involved in the generation of phase III activity of the interdigestive migrating motor complex of the stomach and the gut [32]. Accordingly, higher plasma concentrations of motilin may lead to exaggerated gut motility and probably also to accelerated stomach emptying [32]. Although this is speculative, plasma motilin concentrations showed a positive correlation to the gastrointestinal symptom score in the morning and in the evening but not to the AMS sum score. In rats, however, acute exposure to hypobaric hypoxia at the simulated height of 5000 m decreased plasma motilin levels and inhibited gastric emptying and intestinal propulsion [22]. After the liquid meal, the kinetics of motilin release was comparable at low and high altitude. However, as peptide levels did not differ, the test at Jungfraujoch might have been done too early after ascent.

The hypobaric hypoxic environment caused a marked and significant reduction of fasting and stimulated plasma concentrations of pancreatic polypeptide (PP) within 26 hours. The release of PP is predominantly under vagal cholinergic control [33] but CCK is also able to release PP [34]. Since we found no correlation between fasting levels of PP and CCK, the decrease of PP levels at high altitude seems to represent a reduced basal vagal tone. The significantly lower postcibal plasma concentrations and integrated values of PP at Jungfraujoch could be partly due to reduced plasma levels of CCK, since the stimulated plasma concentrations of the these two peptides were positively correlated. However, so far there are no reports about PP levels at high altitude in humans.

Acute exposure of the subjects to hypobaric hypoxia caused a transient increase of fasting plasma levels of neurotensin which was no longer detectable after 38 hours at high altitude. In contrast, stimulated neurotensin levels significantly decreased from 26 hours onwards at Jungfraujoch. The reason for this divergent behavior of the peptide levels is unclear but was mirrored in the second study after application of the liquid meal: a marked increase of fasting concentrations and suppression of postprandial neurotensin release occurred at high altitude. Experiments with pilots in an altitude chamber showed that irrespective of simulated altitude (208 vs. 1725 m above sea level), acute hypoxia caused a significant increase of fasting plasma levels of neurotensin [15]. Neurotensin is released post-prandially by non-vagal cholinergic pathways from the distal small bowel especially when fat enters the terminal ileum [35]. The observed inhibition of postprandial neurotensin release might thus be due to reduced cholinergic activity.

In the first study we found no influence of the hypobaric hypoxic environment on fasting plasma levels of VIP. In anesthetized dogs, however, portal plasma VIP levels were increased 15 min after the onset of hypoxemia [18]. Responsible for these contradictory findings might be species differences as well as the sites (portal vs. peripheral) and time point of blood sampling. No data are available about postprandial VIP release at high altitude in humans. The source of VIP might be the gastrointestinal tract, namely release from peptidergic vagal fibers inducing pancreatic and intestinal secretion and relaxation of smooth muscles throughout the gastrointestinal tract [36].

In the fasted state we found significant lower plasma levels of ghrelin after acute exposure to hypobaric hypoxic environment. This is in accordance with the results of Shukla et al. [16] (48 h at 3600 m by air). However, chronic hypoxia (7 days at 4300 m [16] or 7 weeks at 5100 m [10] reversed the decrease of ghrelin. Notably, in another study seven days at 2650 m did not alter plasma ghrelin levels in obese subjects [6]. Ghrelin typically increases body weight gain through a stimulation of food intake while decreasing energy expenditure and lipid utilization [37], [38]. Accordingly, our observation that ghrelin shows only a transient decrease at acute exposure to hypoxia suggests that ghrelin does play a major role in the well known anorectic effects of normal [10], [16], [39] and obese [6] subjects exposed to high altitude for longer periods. In accordance with Tschöp et al. [40], postprandial plasma levels of ghrelin showed a transient nadir. This kinetics was not altered at high altitude.

In summary, the enhancement of plasma gastrin levels and the reduction of plasma PP and plasma neurotensin levels strongly suggest a suppression of cholinergic tone, both vagal and non-vagal. This aspect has so far not been considered in the pathophysiological hypothesis of AMS or high altitude illness [1], [2]. A possible explanation could be that endogenous opiates released by stress under hypoxic conditions trigger the inhibition of cholinergic activity. This is based on the observation that the endorphine methionine-enkephalin and the µ-opiate agonist loperamide subtotally block PP release [41], [42].

The initial reduction of fasting plasma CCK is unclear, while postprandial attenuation of CCK release could be due to alterations of gastric emptying and passage of the liquid meal through the upper intestine, the site where CCK is predominantly released [29]. Attenuated CCK release would mean a reduced satiety signal, but fasting and postprandial ghrelin is also reduced at acute hypoxia. Therefore, acute effects of hypoxia on net food uptake might be small. Except motilin with gastrointestinal symptoms, no correlations were detected between peptide levels and AMS symptoms.

In conclusion, acute exposure to hypobaric hypoxic environment exerts no uniform influence on fasting and stimulated plasma levels of GEP peptides in humans. The panel of peptides measured allows to speculate on an acute reduction of the cholinergic tone. However, the mechanisms of AMS development cannot be answered with peptide data.
